# Brazilian Dengue Virus Type 2-Associated Renal Involvement in a Murine Model: Outcomes after Infection by Two Lineages of the Asian/American Genotype

**DOI:** 10.3390/pathogens10091084

**Published:** 2021-08-26

**Authors:** Fernanda Cunha Jácome, Gabriela Cardoso Caldas, Arthur da Costa Rasinhas, Ana Luisa Teixeira de Almeida, Daniel Dias Coutinho de Souza, Amanda Carlos Paulino, Marcos Alexandre Nunes da Silva, Ortrud Monika Barth, Flávia Barreto dos Santos, Debora Ferreira Barreto-Vieira

**Affiliations:** 1 Laboratory of Viral Morphology and Morphogenesis, Instituto Oswaldo Cruz, Fiocruz, Avenida Brasil, 4365, Manguinhos, Rio de Janeiro 2104-90, RJ, Brazil; gabrielacardosocaldas@gmail.com (G.C.C.); rasinhas@protonmail.com (A.d.C.R.); almeida.analuisa98@gmail.com (A.L.T.d.A.); dcoutinho@id.uff.br (D.D.C.d.S.); amandacarlos.bio@gmail.com (A.C.P.); marquinhosans@hotmail.com (M.A.N.d.S.); monikabarth@gmail.com (O.M.B.); barreto@ioc.fiocruz.br (D.F.B.-V.); 2 Laboratory of Viral Immunology, Instituto Oswaldo Cruz, Fiocruz, Avenida Brasil, 4365, Manguinhos, Rio de Janeiro 2104-90, RJ, Brazil; flaviabarretod1@gmail.com

**Keywords:** dengue 2, Asian/American lineages, BALB/c mice, kidney, histopathology and transmission electron microscopy

## Abstract

Dengue virus type 2 (DENV-2) is, traditionally, the most studied serotype due to its association with explosive outbreaks and severe cases. In Brazil, almost 20 years after the first introduction in the 1990s, a new lineage (Lineage II) of the DENV-2 Asian/American genotype emerged and caused an epidemic with severe cases and hospitalizations. Severe dengue includes multiple organ failure, and renal involvement can be potentially related to increased mortality. In order to better understand the role of DENV infection in renal injury, here we aimed to investigate the outcomes of infection with two distinct lineages of DENV-2 Asian/American genotype in the kidney of a murine model. BALB/c mice were infected with Lineages I and II and tissues were submitted to histopathology, immunohistochemistry, histomorphometry and ultrastructural analysis. Blood urea nitrogen (BUN) was detected in blood sample accessed by cardiac puncture. A tendency in kidney weight increase was observed in mice infected with both lineages, but urea levels, on average, were increased only in mice infected with Lineage II. The DENV antigen was detected in the tissue of mice infected with Lineage II and morphological changes were similar to those observed in human dengue cases. Furthermore, the parameters such as organ weight, urea levels and morphometric analysis, showed significant differences between the two lineages in the infected BALB/c, which was demonstrated to be a suitable experimental model for dengue pathophysiology studies in kidneys.

## 1. Introduction

Dengue virus serotypes 1 to 4 (DENV-1–4) are arboviruses belonging to the genus *Flaviviru*s of the family *Flaviviridae* [1]. Transmission occurs in over 125 countries and around 4 billion people are at risk of infection annually [2]. Dengue poses a major threat to urban populations in Asia and Latin America, mainly due to its increased incidence in the last 50 years [3,4,5]. In Brazil, Lineage I of DENV-2 Asian/American genotype has been circulating since the 1990’s [6], when the first cases of dengue haemorrhagic fever and dengue shock syndrome (DHF/DSS) were reported [7]. After 17 years, the emergence of Lineage II of DENV-2 Asian/American genotype was associated with increased disease severity and a high mortality rate, especially in children [6,8,9,10]. However, despite belonging to the same lineage from the 2007–2008 epidemic, DENV-2 strains circulating in 2019 are phylogenetically distant from any Brazilian strain, probably originating in Puerto Rico [11].

Dengue symptoms range from a mild flu-like syndrome to a severe and, sometimes, fatal disease, classified by the World Health Organization (WHO) as severe dengue (SD) which may affect multiple organ systems [12]. The DENV has been detected in a number of organs [13,14,15,16] and, although the liver is the most commonly affected one [17,18], gastrointestinal, hepatic, respiratory, cardiac, neurological and renal manifestations during DENV infection have already been reported [19,20,21,22,23,24,25,26].

The presence of DENV in the kidney has already been demonstrated through the detection of the viral antigen in the tissue cells and in macrophages and monocytes circulating in kidney blood vessels [13,15,17,26,27]. Furthermore, the observation of microtubule reticular structures and dilatation of endoplasmic reticulum in necrotic cells and dense virus-like particles in glomeruli in a transmission electron microscope (TEM), suggested viral infection [17,28,29]. 

Kidney damage induced by DENV infection can result from a direct viral cytopathic effect, inflammatory mediators released in response to the infection, hemodynamic instability, rhabdomyolysis, hemolysis or acute glomerular injury [17,30,31]. Increased levels of urea, creatinine, proteinuria, hematuria, glomerulonephritis and acute DENV infection have been related to dengue [32,33,34,35].

Acute kidney injury (AKI) and acute renal failure (ARF) are significant complications of dengue, and patients presenting SD are more likely to develop them [25,34,36,37,38,39,40]. Rates of mortality due to AKI are 1% for classic dengue, 12–40% for DHF and 60% for DSS [41]. 

Analysis of kidney samples from DENV-infected human cases revealed parenchyma and circulatory damage [17,27]. Tubular necrosis, evidenced by the presence of pyknotic nuclei of epithelial cells, thickening of the glomerular basement membrane, mesangial proliferation, glomerular congestion and hyalinosis, interstitial area with focal fibrosis, diffuse mononuclear infiltrate and hemorrhage foci in the cortical and medullary regions and the increase in populations of CD68+ and CD4+ cells have been reported [27,42,43,44]. 

The pathophysiological basis for SD is yet to be fully understood and likely to be multifactorial [45], involving the host’s genetic background and immunological status, sequence of serotypes in secondary infections, serotype and virulence of infectious viral strains [46,47]. In fact, DENV infecting strain can contribute to detrimental progression of severe disease and death [48,49].

An ideal experimental model for studying dengue should recapitulate the disease progression as it occurs in humans [50]. To date, there are no experimental models that fulfill this requirement [51,52], which hinders the thorough comprehension of DENV pathogenesis mechanisms as well as drug and vaccine development [53]. It is believed that immunocompetent mice are less susceptible to DENV infection [52]. However, studies have shown that BALB/c mice present thrombocytopenia, increased levels of hepatic enzimes alanine aminotrasferase (ASL) and aspartate aminotransferase (ALT), anorexia, weight loss, anemia and even develop severe disease and paralysis, when infected with neuroadapted strains [54,55,56,57]. Viral replication and dissemination have been observed in this experimental model infected with DENV-1, -2 and -4 and viral genome or antigen have been detected in heart, lungs spleen, brain, liver, kidneys and saliva samples [53,54,55,58,59,60,61,62,63]. In kidney tissue of DENV infected BALB/c mice, increased glomerular volume and mesangial cellularity have been reported [58].

Since the DENV infection outcome can be affected by the virulence of different strains [47], and dengue related histopathological data on kidney is still scarce, here we aimed to investigate the renal involvement of BALB/c mice after infection with two distinct DENV-2 Asian/American lineages. Moreover, as this report is a part of a project whose goal is to present BALB/c mice as a suitable non-severe dengue experimental model for studies on the pathogenesis of dengue in different organs, we have recently demonstrated the susceptibility of the aforementioned murine model to both lineages and showed that the changes induced by those strains in the liver were similar to those observed in human cases, but it was observed that some parameters were manifested at different times of infection [63].

## 2. Results

### 2.1. Evaluation of Kidney Weight

**Figure 1:**
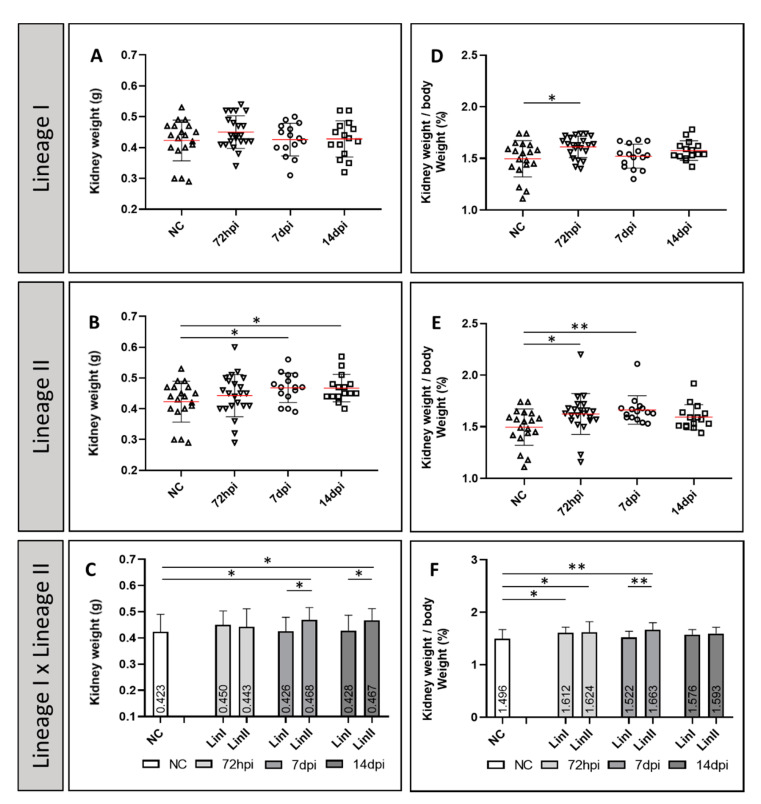
Mean kidney weight (**A**–**C**) and weight/body weight ratio (%) (**D**–**F**) of BALB/c mice uninfected and infected with DENV-2 Strains 72 hpi, 7 and 14 dpi. CN: (*n* = 19); LinI: 72 hpi (*n* = 22), 7 dpi (*n* = 15), 14 dpi (*n* = 15); LinII: 72 hpi (*n* = 22), 7 dpi (*n* = 15), 14 dpi (*n* = 15). NC: negative control, *n*: number of mice, hpi: hours post-infection, dpi: days post-infection, Lin: Lineage, *: *p* < 0.05, **: *p* < 0.01.

A slight increase in the mean weight of the kidney of infected mice was observed when those were infected by both Lineages of DENV-2 Asian/American genotype, when compared to the uninfected mice (mean = 0.423 g/standard deviation (±) = 0.06). The mean weight of kidneys of mice infected by Lineage I presented its highest value at 72 hpi and subsequent weight loss at 7 dpi and 14 dpi (from 0.45 ± 0.05 g to 0.426 ± 0.05 g and 0.428 ± 0.05 g, respectively). In individuals infected by Lineage II, there was an increase in the mean organ weight at all times of infection: at 72 hpi, the mean weight was 0.43 ± 0.06 g, at 7 dpi, 0.468 ± 0.04 g and at 14 dpi, 0.477 ± 0.04 g. The difference between the means of individuals infected with Lineage I and Lineage II was statistically significant at 7 and 14 dpi (*p* = 0.03 and 0.048, respectively) and, the difference between Lineage II and uninfected mice was statistically significant at 7 and 14 dpi (*p* = 0.035 and 0.034, respectively) (Figure 1A–C). Likewise, the means of the ratio of the kidney weight to the total body weight of infected mice by both Lineages of DENV-2 Asian/American genotype, increased, on a small scale, when compared to the uninfected controls (mean = 1.496 ± 0.17%) at all times of infection. In Lineage I infected mice, the average percentage (1.621 ± 0.01%) was significantly higher than the observed for the uninfected control group at 72 hpi (*p* = 0.013), decreasing after the 7 dpi (1.522 ± 0.11%) and rising again, on the 14 dpi (1.576 ± 0.09%). In Lineage II infected mice, the highest ratio between the kidney and the body weight (mean = 1.663 ± 0.19%), was also statistically significant (*p* = 0.005) when compared to the uninfected control group, but it was observed at 7 dpi. Mice infected with this Lineage, and euthanized at 72 h and 14 dpi, also had higher means than those for the uninfected mice (1.624 ± 0.17% and 1.593 ± 0.12%, respectively) (Figure 1D–F). In addition, there was a statistically significant difference between the uninfected control group and the groups infected by Lineages I at 72 hpi (*p* = 0.013) and II at 72 hpi (*p* = 0.005) and at 7 dpi (*p* = 0.005); and between the infected groups at 7 dpi (*p* = 0.005) (Figure 1D–F).

### 2.2. Evaluation of Urea (BUN) Levels

**Figure 2:**
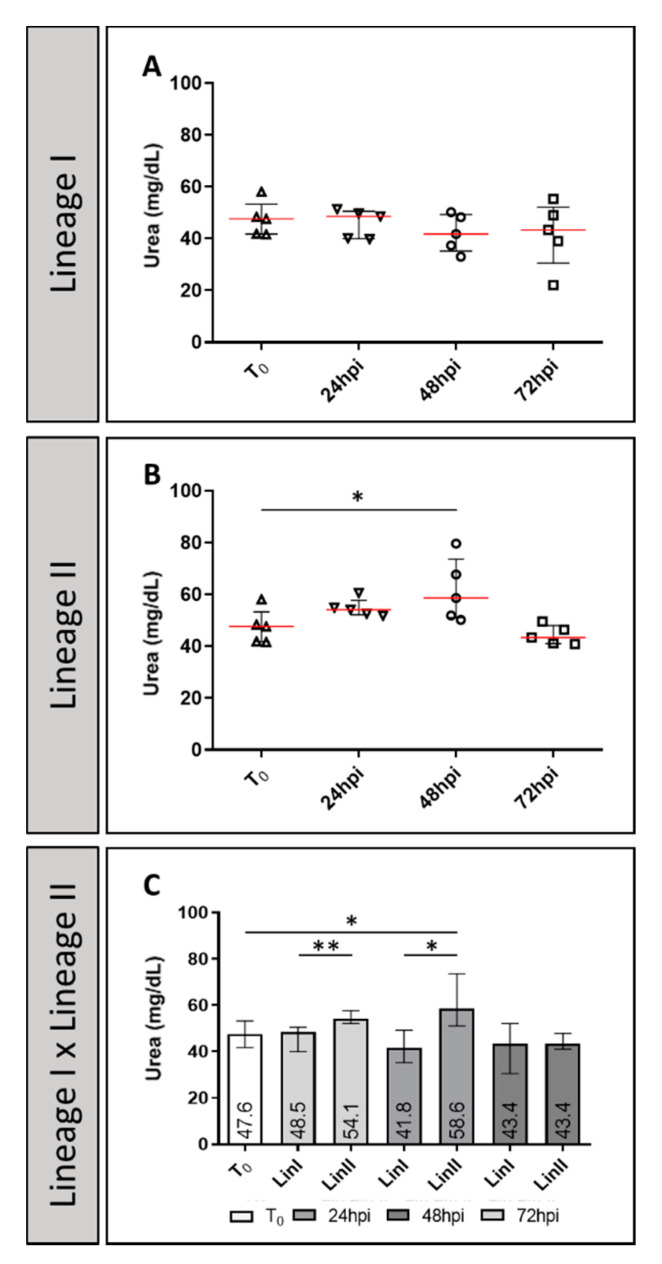
Urea levels (mg/dL) of BALB/c mice uninfected and infected with DENV-2 Lineages 24 hpi, 48 hpi and 72 hpi. CN: (*n* = 5); LinI: 24 hpi (*n* = 5), 48 hpi (*n* = 5); 72 hpi (*n* = 5); LinII: 72 hpi (*n* = 5); 7 dpi (*n* = 5); 14 dpi (*n* = 5). N: number of mice, hpi: hours post-infection. NC: negative control, Lin: Lineage, *: *p* < 0.05, **: *p* < 0.01.

The concentration of BUN in serum of uninfected mice control group was 47.6 mg/dL and interquartile range (IR) = 41.9–44.8). The medians presented for Lineage I were 48.5 mg/dL (IR = 40.1–49.7) at 24 hpi; 41.8 mg/dL (IR = 37.4–48.3) at 48 hpi and 43.4 mg/dL (IR = 39–49) at 72 hpi. In Lineage II infected mice, the level of the organic compound present in the serum, rose on the first day of infection (median = 54.1 mg/dL, IR = 52.5–54.9) and peaked at 48 hpi (median = 58.6 mg/dL, IR = 51.9–67.7). At 72 hpi, BUN concentration (median = 43.4 mg/dL, IR = 41.1–46.4) was lower than that of the uninfected mice control group. The differences between the medians were statistically significant between negative control and Lineage II at 48 hpi (*p* = 0.028) and between the two DENV-2 Lineages at 24 and 48 hpi (*p* = 0.009 and 0.011, respectively) (Figure 2).

### 2.3. Evaluation of Histopathological Alterations, Histomorphometry and Antigen Detection

**Figure 3:**
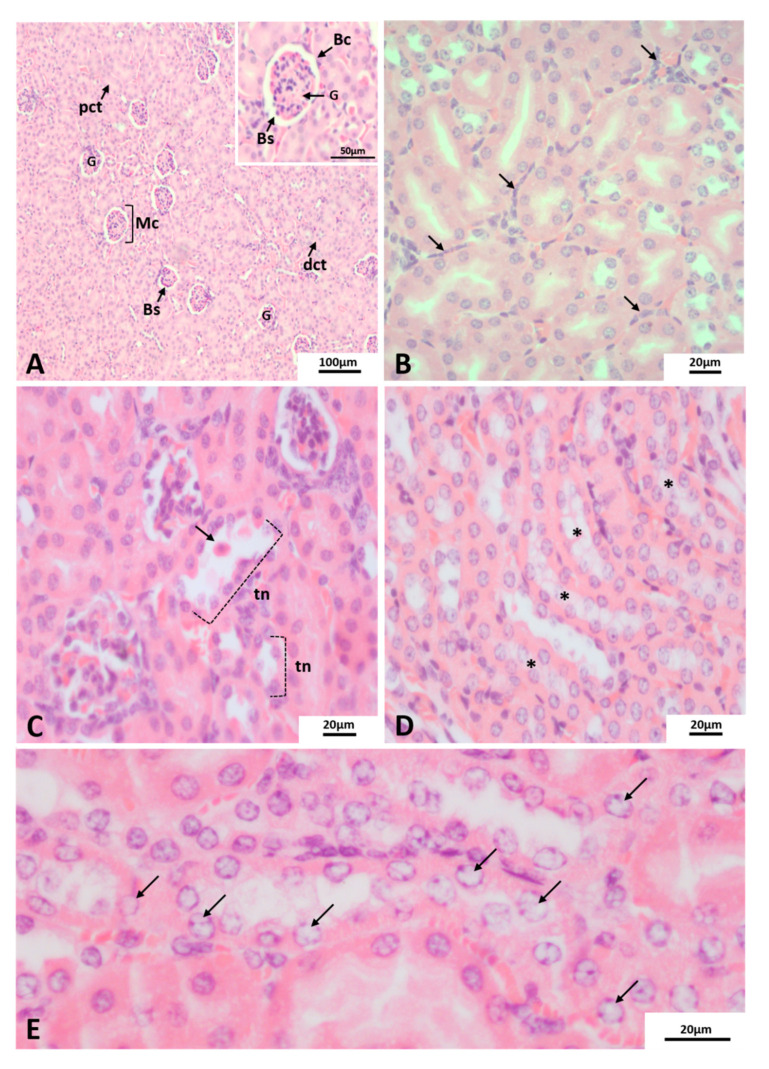
Histopathological alterations of renal cortex of BALB/c mice. H and E staining. Euthanasia; 72 hpi. (**A**) non-infected mice. (**B**–**D**) mice infected with DENV-2 Lineages. (**B**) mononuclear cell infiltrate, (**C**) tubular necrosis (tn), desquamation of necrotic cells (arrow). (**D**) areas of cytoplasmic loss (*) (**E**) chromatin loss [arrows]. Mc: Malpighian corpuscle, G: glomerulus, dct: distal convoluted tubules pct: proximal convoluted tubules. Bs: Bowman’s space, Bc: Bowman capsule. Experimental infection: (**C**,**E**) Lineage I, (**B**,**D**) Lineage II.

Kidney tissues of uninfected mice presented well preserved Malpighian corpuscles (Mc), composed by Bowman capsule and glomeruli. There were no signs of atrophy, glomerulitis or bleeding. In addition, the interstice, proximal and distal contorted tubules and collecting tubules showed no signs of exudate, inflammatory infiltrates or hemorrhage (Figure 3A). The images displayed herein are representative of alterations observed in tissues of BALB/c mice infected with either Lineage I or II and euthanized at 72 hpi.

**Figure 4:**
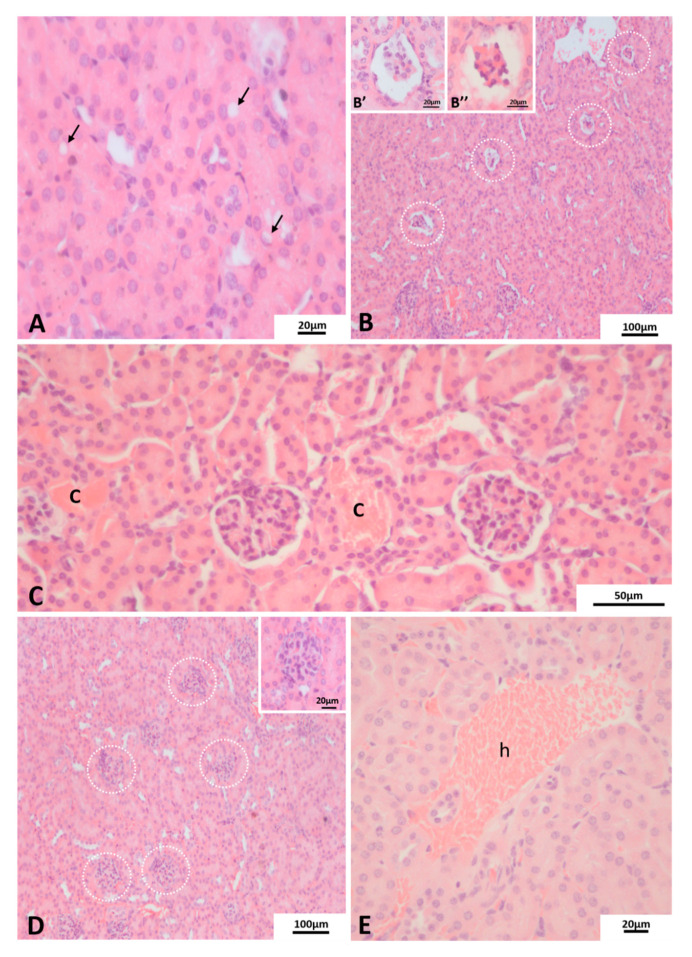
Histopathological alterations of renal cortex of BALB/c mice. H and E staining. Euthanasia; 72 hpi. (**A**) cytoplasmic inclusions (arrows). (**B**) glomerular atrophy (circled/insets). (**C**) congestion (C), (**D**) enlargement of glomerular volume (circled area/inset), (**E**) focal hemorrhage (h). Experimental infection: (**A**,**B**,**B’**,**B’’**) Lineage I, (**C**–**E**) Lineage II.

The morphological changes observed in kidney tissue of BALB/c mice infected by the two DENV-2 Lineages were focal and did not differ qualitatively. Mononuclear inflammatory cells infiltrate (Figure 3B), peritubular congestion (Figure 4C) and tubular necrosis (Figure 3C–E) were the most common histopathological changes observed among samples. In addition to desquamation of necrotic cells and loss of microvilli of cubic epithelium that constitutes the convoluted tubules (Figure 3C), high chromatin (Figure 3E) and cytoplasmic (Figure 3D) loss was observed. Those apparently vacuolated cells, when present in increased amount and clustered, gave the cortical parenchyma, a translucid appearance. Moreover, a number of tubular cells presented regular cytoplasmic inclusions (Figure 4A).

The commonest alteration observed in renal corpuscle was glomerular atrophy (Figure 4B), presented as an apparent reduction in the number of cells that constitute the glomerulus (Figure 4B’) or with the glomerulus forming a compact and reduced cell mass (Figure 4B’’). In the latter, it was possible to notice the loss of integrity of the parietal layer of Bowman’s capsule, and in both cases, an increase in Bowman’s space was noted. Furthermore, in some renal cortex regions, it was not possible to distinguish the Bowman’s space from the renal corpuscle due to enlargement of glomerular area caused by apparent cellularity increase (Figure 4D). Finally, focal hemorrhage (Figure 4E), a common DENV infection feature, was observed tissues from infected BALB/c with both DENV-2 lineages. Table 1 shows the number of mice in which each alteration was observed.

**Figure 5:**
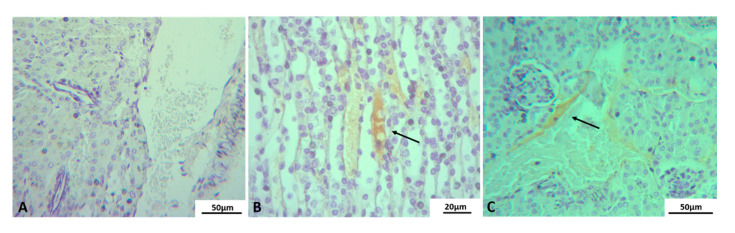
DENV antigen detection in kidney of BALB/c mice infected with DENV-2 Lineage II. Euthanasia: 72 hpi. (**A**) Negative control showing no peroxidase reactive cells, (**B**) peroxidase reactive epithelial cells from the loop of Henle (arrow), (**C**) peroxidase reactive endothelial cells (arrow). Experimental infection: Lineage II.

As expected, no DENV antigen staining was observed in the kidney tissue of the uninfected mice control group (Figure 5A). However, samples from Lineage II infected BALB/c, viral staining was observed in epithelial cells in the medullary area (Figure 5B) and endothelial cells in the cortical area (Figure 5C). 

**Figure 6:**
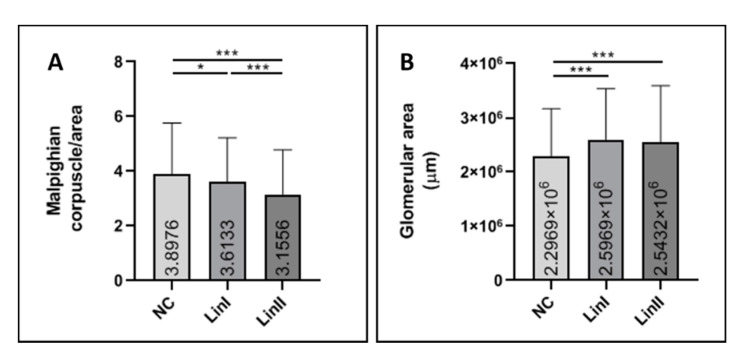
Malpighian corpuscles count (**A**) and mean area occupied by glomeruli (**B**) of BALB/c mice infected with DENV-2 Lineages. Euthanasia; 72 hpi. Glomerular area standard deviation: NC (±8.69 × 10^5^), Lin I (±9.41 × 10^5^), Lin II (±1.04 × 10^6^). NC: negative control, Lin: Lineage. *: *p* < 0.05, ***: *p* < 0.00.

At 72 hpi, a statistically significant decrease in the number of corpuscles in mice infected with both Lineage I (*p* = 0.016) and Lineage II (*p* ≤ 0.001) was observed when those were compared to uninfected BALB/c. In the latter, it was possible to observe 3.89 (±1.8) Mc per analyzed area. For mice infected with DENV-2 Lineages I and II, the averages were 3.61 (±1.6) and 3.15 (±1.6) Mc per analyzed area, respectively. The difference in the number of renal corpuscles between the two DENV-2 Lineages was also statistically significant (*p* ≤ 0.001) (Figure 6A). The mean area occupied by glomeruli was measured and, despite the decrease in the number of Mc and glomerular atrophy observed in the infected mice, the mean area occupied by glomeruli in mice infected with DENV-2 exceeded the control group (*p* ≤ 0.001 for both strains) (Figure 6B).

### 2.4. Evaluation of Ultrastructural Alterations

**Figure 7:**
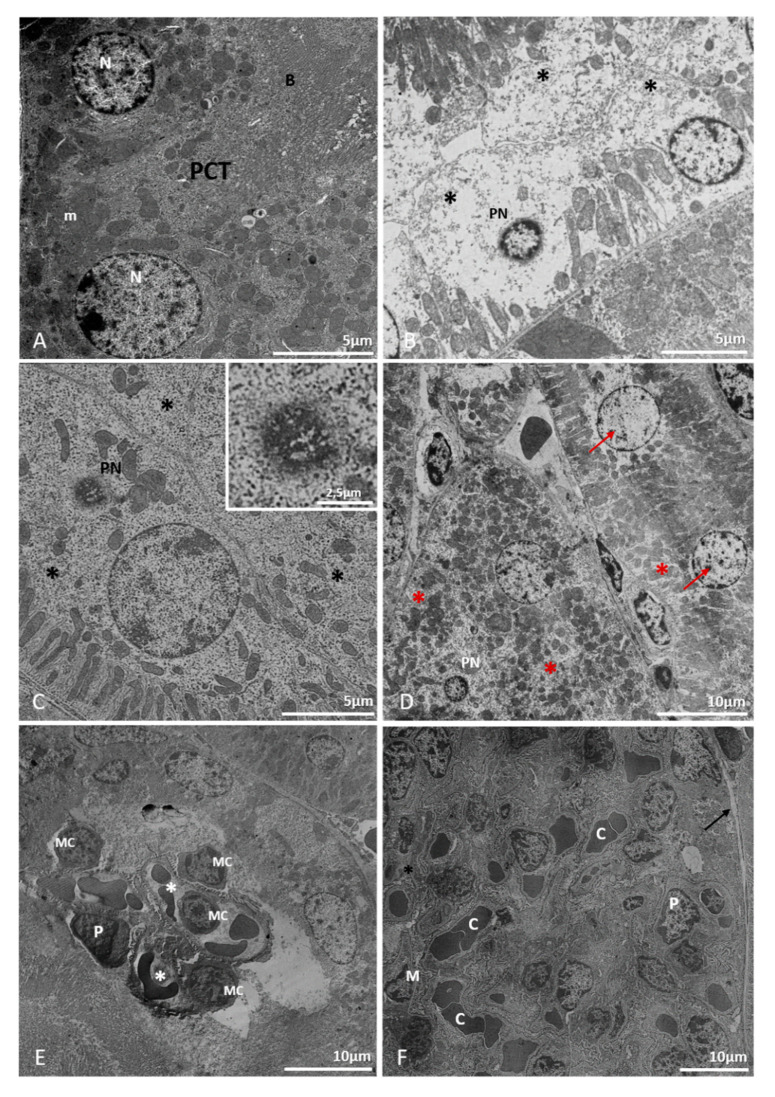
Electron micrography of kidney samples of BALB/C mice. (Noninfected: A; infected with DENV-2 Lineage I or II: (**B**–**E**)). (**A**): proximal convoluted tubule (PCT), brush border (B), nucleus (N), mitochondria (m). (**B**): pyknotic nuclei (PN), massive cytoplasmic loss (*). (**C**): pyknotic nuclei (PN/arrow), cytoplasmic rarefaction (*). (**D**): pyknotic nuclei (PN), chromatin loss (arrow). cytoplasmic rarefaction (*), (**E**): glomerular mononuclear infiltrate. Mononuclear cell (MC), podocyte (P), capillary (*), (G) glomerulus, (**F**): capillary congestion. (**C**), podocyte (P), mesangial cell (M), mononuclear cell (MC), reduced Bowman space [arrow]. Experimental infection: Lineage I (**C**,**E**), Lineage II (**B**,**C**,**F**).

The ultrastructural analysis of kidney tissue of uninfected and infected BALB/c mice euthanized at 72 hpi, corroborates histopathological findings. As expected, tissues from uninfected control mice showed no damage to the renal parenchyma. Epithelial cells presented round nuclei with regular looking chromatin pattern and no cytoplasmic rarefaction or pyknotic nuclei were observed (Figure 7A).

The renal tissue of DENV-2 infected mice showed cytoplasmic rarefaction (Figure 7B–D), pyknosis (Figure 7B,C) and death of convoluted tubule’s epithelial cells (Figure 7B), which are consistent with tubular necrosis. Additionally, a number of epithelial cells presented altered distribution and amount of chromatin in the nucleus, which could be caused by a process of karyolysis (Figure 7D).

**Figure 8:**
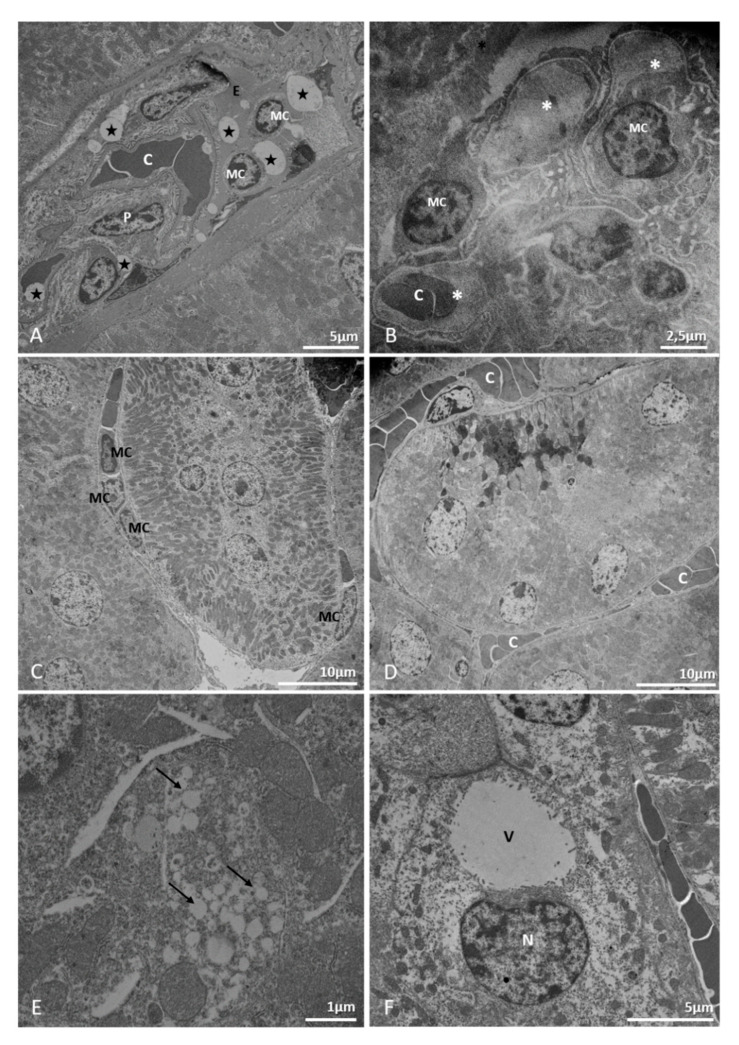
Electron micrography of kidney samples of BALB/C mice infected with DENV-2 Lineage I or II. (**A**,**B**): congested glomeruli. Capillary congestion (C), edema [E], vesicles within glomerulus [star], capillary (*), mononuclear cell (MC). (**C**): mononuclear cell infiltrate (MC). (**D**): capillary congestion. (**E**): lipid-like cytoplasmic inclusions (arrows). (**F**): nucleus (N) dislocated by vesicle (V). Experimental infection: Lineage I (**B**,**C**), Lineage II (**A**,**D**–**F**).

Mononuclear inflammatory cells infiltrates were present both in renal interstitium (Figure 7E) and within glomeruli (Figure 8A,B). Some glomeruli appeared to be congested due to increase in cellularity and edema, and as observed in our histological sections, Bowman’s space could not be distinguished (Figure 7F and Figure 8A). Moreover, focal areas in the renal cortex were congested (Figure 8D) and small vesicles (Figure 8E) and inclusions of unknown nature were observed within epithelial cells (Figure 8F) and in the edematous area of a glomerulus (Figure 8A).

## 3. Discussion

In Brazil, the emergence of the Lineage II DENV-2 in 2007–2008 resulted in major outbreaks with a new epidemiological profile and number of severe cases, hospitalizations and deaths, especially in children 15 years old and under [6,8,9,10,64]. Moreover, it has been shown that genetic variations are important determinants of viral fitness, virulence and tropism [47,59,65].

Although some authors suggest that the kidney is not a target organ of DENV due to the lack of evidence of viral replication [13,17], renal involvement during dengue is fairly well documented. Proteinuria, hematuria and glomerulonephritis have been reported during or shortly after acute DENV infection. Moreover, AKI and ARF are somewhat common features among patients with DHF/DSS [22,34,35,43,66,67,68,69]. Because data on renal manifestations induced by DENV infection are not as abundant as data concerning target organs, in this study, we sought to characterize and compare the alterations induced by two distinct DENV-2 Lineages of the Asian/American genotype in the kidney of BALB/c mice.

Our results showed that there was a tendency of kidney weight increase in some infected mice when compared to uninfected control group. To ensure that the increase in kidney weight was not solely a result of body weight gain, the kidney weight/body weight ratio (%) was calculated, and means of the infected group were also higher than control group. Despite that, to our knowledge, there are no reports of kidney weight increase due to DENV infection, liver, spleen and pancreas weight increases during DENV infection, and hepatomegaly, splenomegaly and pancreatic enlargement have been associated with dengue [70,71,72,73]. 

Altered vascular permeability, accompanied by plasma and albumin leakage, is a common feature of dengue pathophysiology [5], organ enlargement or increase in weight could be a consequence of fluid accumulation in the interstice. We did not observe interstitial edema in the renal tissue analyzed here, only fluid leakage inside glomeruli; however, another study on the same mice model infected by DENV-3, also carried out by our group, showed transudate in the kidney’s cortical area and reported statistically significant increase in kidney weight [74]. Furthermore, histopathological findings on autopsies of human fatal cases also showed edema, although mostly in the medullary region [17,75]. 

For each strain, the weight means reached higher values at distinct times of infection. Lineage I peaked at 72 hpi, while Lineage II peaked at 7 dpi. Difference between the two Lineages was statistically significant at 7 and 14 dpi. A possible explanation for such difference is that Lineage II takes longer to manifest its signs. 

Since urea is reabsorbed by the kidney, its altered levels in the blood may indicate renal dysfunction. Increased levels of BUN are often reported is renal involvement during DENV infections [22,34,37,76,77], and can be a consequence of glomerular injury or hypotension [37]. Mice blood samples were collected at 24, 48 and 72 hpi, and BUN levels were measured. While the median of the Lineage I infected mice was higher than the control group median only at 24 hpi, Lineage II infected mice presented higher BUN levels when compared to those of the uninfected group, at 24 and 48 hpi. Increased levels of BUN in the sera of BALB/c mice infected by DENV-3, supports our findings [74]. Furthermore, difference between Lineage II and control group was statistically significant at 48 hpi and between Lineages I and II infected mice, at 24 and 48 hpi, and this difference may be due to the different viral strains or host genetic factors.

The morphological changes described in this study were from kidney of mice infected with each one of the DENV-2 Lineages and euthanized 72 hpi. The alterations observed in renal tissues were focal and did not differ qualitatively. On the other hand, quantitatively, the difference between Lineages I and II, regarding enlargement of glomeruli (90% and 60% of infected mice, respectively) was noteworthy. In accordance with these results, morphometric analysis revealed that, on average, glomerular area in mice infected with the Lineage I slightly exceeded the glomerular area in mice infected with by Lineage II. Nonetheless, the difference was not significant.

Glomerular changes affecting the kidney are often reported in dengue human cases [31,34,43] and, DENV inducing glomerulopathies is well documented [34,67,77,78]. Deposition of immuno complexes has been suggested as a mechanism of glomerular injury in AKI induced by dengue [28]. Moreover, it has been suggested that glomerulonephritis results from an autoimmune mediated glomerular damage triggered by the virus [79].

In this study, renal tissue of DENV-2 infected BALB/c mice presented both glomerular atrophy and enlargement of glomeruli. Indeed, the morphometric analysis showed that, while, on average, the glomerular area of infected mice exceeded that of the uninfected control group, Mc/glomeruli on uninfected mice kidneys outnumbered the ones in infected groups. Our findings on Mc counting could suggest that the atrophy is an early stage of necrosis, since, in a similar study, Caldas that observed glomeruli in different stages of atrophy, as well as, areas free of glomeruli in the renal cortex of BALB/c mice infected with DENV-3. Difference of Mc number was statistically significant among all groups.

We observed areas with enlarged glomeruli, due to increased cellularity, to the point of Bowman’s space obliteration. By TEM, it was possible to observe that the enlargement was a result of mesangial proliferation and mononuclear cells migration. Analysis of tissues from dengue patients and BALB/c mice experimentally infected has associated mesangial proliferation with deposition of immuno complexes in glomeruli [33,58,80,81]. The increase in the cellularity resulted in congested capillaries and edema. Nunes [27] and Pagliari [43] also observed congestion in capillaries in glomeruli; however, no mesangial proliferation was reported. 

One of the hallmarks of dengue pathogenesis is the involvement of the endothelium [82]. Vascular permeability plays an important role in SD pathogenesis [83,84]. Our ultrastructural studies showed some fluid leakage in the glomeruli, but we did not observe any endothelial damage. The capillary permeability could be due to the release of inflammatory mediators from the mononuclear cell present, both inside capillaries and among mesangial cells, in the glomeruli. This hypothesis is supported by authors who believe that altered vascular permeability in dengue is caused by immunological host response, rather than by infection of endothelial cells or cell death [84,85,86]. Moreover, our histopathological findings showed hemorrhage foci and small mononuclear cells infiltrates in the cortical area of kidney of the DENV-2 infected mice and corroborates studies carried out with human cases of DENV-3 and -4 infection, and BALB/c mice infected with DENV-3 [17,27,74,75,87]. 

Studies on human autopsy tissues have described tubular necrosis on dengue cases [17,27,43]. Mohsin [76] reported a case of ARF with tubular necrosis in a dengue patient who did not present signs of hemorrhagic fever, only classic dengue symptoms. Moreover, it has been suggested that necrosis results from ischemic processes due to severe hypovolemic shock, hypoprofusion and hypoxia which leads to decreased kidney perfusion, interstitial edema and mononuclear infiltration, and acute glomerulonephritis [17,76,88,89]. In a BALB/c model, Caldas [74] observed mitotic figures, indirect signs of hyperplasia, which may be related to tubular injury.

In this study, all kidneys of DENV-2 infected mice presented tubular necrosis, mostly in proximal convoluted tubules. The tissues showed desquamation of epithelial cells and loss of the border brush. Conversely, Caldas [74] observed the thickening of the brush boarder. Autopsy data described loss of basement membrane, pyknotic nuclei and dilation of endoplasmic reticulum in necrotic cells; tubular hemorrhage and atrophy with discrete mononuclear inflammatory infiltrate. Besides that, IL18 and IL6, both proinflamatory citokines, were detected in tubular cells [17,27,43,75]. Here, some nuclei were pyknotic, an alteration seen in the same murine model infected by DENV-3 [74]. Others, presented massive chromatin loss. There were areas of cytoplasmic rarefaction and some tubular epithelial cells were lightly stained, even though the plasmatic membrane looked intact. Upon ultrastructural analysis, it was possible to see that those cells had lost most of its contents and cytoplasm was almost completely absent.

Additionally, histological analysis revealed some dislocated nuclei due to fairly large unstained round cytoplasmic inclusions resembling lipid droplets. These inclusions were also seen by Caldas [74]. In fact, samples presented small lipid-like inclusions, however, the structures responsible for nuclei dislocation were, actually, vesicles that, to our knowledge, has not been described as an alteration in renal tissue during dengue.

DENV-like particles have been observed in ultrathin sections of BALB/c mice infected by DENV-3 and tissues of renal biopsy [28,74], but they were not observed here. At 72 hpi, two out of 10 kidneys of the infected mice were positive for the viral antigen, both of them, from mice infected by Lineage II, and morphological changes compatible with those reported both in human cases and animal models of infection [17,58,69,74,75,90] were also observed in this study. The detection of anti-DENV antigen, in tubular cells and infiltrate macrophages and monocytes, has been reported in studies on human cases and experimental models [13,15,17,27,90,91], however, no virus RNA negative strand was detected and, NS3 was only detected by Nunes [27] in mesangial cells and macrophages. Even though some studies report viral RNA detection in kidneys of human biopsies and animal models tissues [15,27,74,90], some authors hypothesize that DENV antigens detected in the kidney are from reabsorbed immuno complexes, and that it is more likely that damage to renal parenchyma is caused by immuno-mediators released as a host response to DENV infection [17,43,44], or secondary to other dengue complications, such as rhabdomyolysis, myositis, hypoperfusion and hypoxia [37,42,66,92,93,94].

Several studies have reported DENV infection leading to kidney injury, especially in SD. Mortality rates among DHF patients who develop AKI can reach up to 60% [89]. Therefore, the mechanisms behind renal involvement in dengue must be better understood. Our results show that BALB/c mice infected with two distinct Lineages of DENV-2 Asian/American genotype presents renal alterations that are commonly observed in human cases and may be a suitable experimental model for studies of pathophysiology and immunopathogenesis of dengue in kidney.

## 4. Material and Methods

### 4.1. Ethical Statement

All experiments with mice were conducted in compliance with Ethical Principles in Animal Experimentation stated in the Brazilian College of Animal Experimentation and approved by the Institute’s Animal Use Ethical Committee (L-023/2018) and the Human Research Ethic Committee (274/05) from the Oswaldo Cruz Institute (IOC), Oswaldo Cruz Foundation (FIOCRUZ).

### 4.2. DENV-2 Viral Strains

DENV-2 strains BR/RJ66985/2000 (GenBank #HQ012518) and BR/RJ0337/2008 (GenBank #HQ01253), representative of Lineage I and Lineage II from the Asian/American genotype [6], were isolated from patient sera at the Flavivirus Laboratory, IOC, FIOCRUZ, during the epidemics of 2000 and 2008, respectively, and kindly provided. Serotype was confirmed by indirect immunofluorescence, using DENV-type-specific monoclonal antibody (3H5), and RT- PCR [95,96]. Viral stocks were prepared by inoculating 100 µL of each strain into 175 cm^2^ cell culture bottles containing mosquito Aedes albopictus cell line (C6/36) at a concentration of 5 × 10^5^ cells/mL. Titers of both strains (BR/RJ66985/2000: 10^6.66^ TCID_50_/0.1 mL and BR/RJ0337/2008: 10^9^ TCID_50_/0.1 mL) were calculated by the Reed and Muench method [97]. The viruses did not undergo any passages through mice brain for neuroadaptation.

### 4.3. BALB/c Experimental Infection

For experimental infection, two-month-old, male BALB/c mice, provided by Institute of Science and Technology in Biomodels (ICTB)-Fiocruz, were used. During experimentation period, the animals were kept under controlled temperature, photoperiod, nutrition and hydration conditions, as previously described [26]. Briefly, for infection with both Lineages I and II of DENV-2, BALB/c mice were inoculated by the intravenous route (IV) through the caudal vein. Inocula volume was 100 µL and viral concentration, 10,000 TCID_50_/0.1 mL. The mice were anesthetized [ketamine = 150 mg/kg, xylazine = 10 mg/kg and tramadol = 10 mg/kg] and euthanized 24, 48 and 72 h post-infection (hpi), at 7 or 14 days post-infection (dpi), according to their experimental group. In order to collect blood samples, cardiac puncture was performed before euthanasia. Kidney samples, destined to morphological and immunohistochemistry analysis, were fixed in Millonig buffered formalin. All kidney samples were weighted immediately after harvesting at 72 hpi, seven and 14 dpi. Non-infected mice were used as negative controls. Table 2 shows the number of mice used in the study.

### 4.4. Biochemical Analysis

For each DENV-2 Lineage, a total of 15 mice were infected. The mice were divided into three groups of five animals, and each group euthanized at different times after infection (24, 48 and 72 hpi). After the determined periods of infection, the mice were anesthetized and blood was collected by cardiac puncture. Blood samples were then centrifuged for 10 min, at 5000 rotations per minute, to separate the serum from the cellular components. Non-infected mice (*n* = 5) blood was collected at the same day as the 72 hpi group. Blood levels of urea were measured by dry chemistry testing using the Vitros 250 equipment (Ortho clinical Diagnostics, Jonhson & Jonhson, New Brunswick, NJ, USA) in collaboration with ICTB.

### 4.5. Bright Field Microscopy

For each DENV-2 Lineage, 10 mice were infected. Five non-infected mice were used as negative control. Seventy-two hpi, the mice were euthanized, kidney samples were collected and fixed in Millonig buffered formalin (formalin P.A.: 100 mL, distilled water: 900 mL, NaH_2_PO_4_: 18.03 g, NaOH: 4.2 g). The samples were then dehydrated in decreasing concentrations of ethanol, clarified in xylene and embedded in paraffin. Tissue sections 5 µm thick were obtained using a microtome (Leica 2025) and stained with hematoxylin and eosin (H and E) and analyzed using a bright field microscope (AxioHome, Carl Zeiss, Oberkochen, Germany). All procedures were performed in collaboration with the Pathology Laboratory, IOC, FIOCRUZ.

### 4.6. Immunohistochemistry

For DENV antigen detection in kidney tissue an immunohistochemistry assay was performed. Briefly, five slides containing histological sections of kidney infected with each DENV-2 lineage were heated at 60 °C for one hour, de-paraffinized in xylene and rehydrated with alcohol. Antigen retrieval was performed by heating the tissue in the presence of citrate buffer. Next, tissues were blocked for endogenous peroxidase with 3% hydrogen peroxidase in methanol for 10 minutes and rinsed in tris-HCl (pH 7.4). To reduce non-specific binding, sections were incubated in Protein Blocker solution (Spring Bioscience, Pleasanton, CA, USA) for 10 min at room temperature. Tissues were incubated with rabbit anti-dengue 4G2 antibody (1:200), used as the primary antibody, and afterwards, with a rabbit anti-mouse IgG-HRP conjugate (REVEAL polyvalent HRP, Spring Bioscience, Pleasanton, CA, USA). Finally, the slides were counterstained with Harris hematoxylin, and analyzed using a bright field microscope (AxioHome, Carl Zeiss, Oberkochen, Germany). Samples from non-infected mice were used as negative control.

### 4.7. Histomorphometry

Morphometrical analysis goals were quantifying the number Malpighian corpuscles (Mc) and measuring the area occupied by glomeruli in kidney samples of each group of mice. Fifteen histological sections of kidney of BALB/c mice euthanized at 72 hpi stained with H and E (5 from non-infected mice as negative control, 5 from mice infected with Lineage I of DENV-2 and 5 from mice infected with Lineage II of DENV-2) were analyzed. For each section, 30 images of random areas were captured at a 200× magnification using a digital camera coupled to a bright field microscope (AxioHome, Carl Zeiss, Oberkochen, Germany). The analyses were performed with the aid of the image processing program Image J. Aiming at the recognition and calculation of the area occupied by the structure, glomerular area was delimited and colored differently from the colors present in the studied field. The values obtained were then compiled by group and the mean was calculated.

### 4.8. Transmission Electron Microscopy (TEM)

Kidney tissues were processed as described by Barreto-Vieira [98]. Briefly, samples were fixed by immersion in 2% glutaraldehyde diluted in sodium cacodylate buffer (0.2 M, pH 7.2), cut into smaller fragments (~1 mm^3^), post-fixed in 1% osmium tetroxide and dehydrated in increasing concentrations of acetone. Subsequently, samples were embedded in Epoxy resin (Electron Microscopy Sciences, Hatfield, PA, USA). For light microscopy, semithin sections (0.5 μm) were stained with methylene blue and azure II and analyzed using a Zeiss PrimoStar light microscope (Carl Zeiss, Oberkochen, Germany). Ultrathin sections were stained with uranyl acetate and lead citrate and analyzed using a Hitachi HT 7800 transmission electron microscopy (Hitachi, Tokyo, Japan).

### 4.9. Statistical Analysis

A database with data related to organ weight, histomorphometry and biochemical analysis was created in Microsoft Excel. The graphs were created using the GraphPad Prism software version 8.0.1. For statistical analysis, a *t*-test was performed when groups presented normal distribution and a Mann–Whitney test was performed when groups presented non-normal distribution, using the SPSS Statistics software version 25. Results of *p* ≤ 0.05 were considered statistically significant.

## Figures and Tables

**Table 1:** Histopathological alterations observed in kidney samples of BALB/c infected with DENV-2 Lineages I or II and euthanized at 72 hpi. Number of mice whose livers presented the alteration/total number infected mice.

Alterations	DENV-2		
	Lineage I (%)	Lineage II (%)	Total (%)
Tubular necrosis	10/10 (100)	10/10 (100)	20/20 (100)
Mononuclear cell infiltrate	9/10 (90)	10/10 (100)	19/20 (95)
Cytoplasmic loss	10/10 (100)	8/10 (80)	18/20 (90)
Capillary congestion	8/10 (80)	9/10 (90)	17/20 (85)
Glomerular atrophy	8/10 (80)	8/10 (80)	16/20 (80)
Chromatin loss	8/10 (80)	7/10 (70)	15/20 (75)
Enlargement of glomeruly	9/10 (90)	6/10 (60)	15/20 (75)
Cytoplasmic inclusions	3/10 (30)	3/10 (30)	6/20 (30)
Haemorrhage	1/10 (10)	3/10 (30)	4/20 (20)

**Table 2:** Number of mice used for histopathological, immunohistochemical and biochemical analysis and measuring of kidneys’ weight.

*n* = 123 Mice	Histopathology/IHQ	TEM	Biochemical Analysis			Kidney Weight		
	72 hpi	72 hpi	24 hpi	48 hpi	72 hpi	72 hpi	7 dpi	14 dpi
DENV-2/Lineage I	10/5	5	5	5	5	22	15	15
DENV-2/Lineage II	10/5	5	5	5	5	22	15	15
Negative control	5/5	5			5			19
Total [samples]	25	15	35			123		

IHQ: immunohistochemistry, TEM: transmission electron microscopy, hpi: hours post infection, dpi: days post infection.

## Data Availability

Not applicable.

## References

[1] Gubler D.J. (2002). Epidemic dengue/dengue hemorrhagic fever as a public health, social and economic problem in the 21st century. Trends Microbiol..

[2] Stanaway J.D., Shepard D.S., Undurraga E.A., Halasa Y.A., Coffeng L.E., Brady O.J., Hay S.I., Bedi N., Bensenor I.M., Castañeda-Orjuela C.A. (2016). The global burden of dengue: An analysis from the Global Burden of Disease Study 2013. Lancet Infect Dis..

[3] Kyle J.L., Harris E. (2008). Global Spread and Persistence of Dengue. Rev. Microbiol..

[4] World Health Organization (2012). Global Strategy for Dengue Prevention and Control.

[5] Guzman M.G., Gubler D.J., Izquierdo A., Martinez E., Halstead S.B. (2016). Dengue infection. Nat. Rev. Dis. Primers.

[6] Faria N.R., Nogueira R.M., de Filippis A.M., Simões J.B., Nogueira F., da Rocha Queiroz Lima M., dos Santos F.B. (2013). Twenty years of DENV-2 activity in Brazil: Molecular characterization and phylogeny of strains isolated from 1990 to 2010. PLoS Negl. Trop. Dis..

[7] Nogueira R.M., Miagostovich M.P., Lampe E., Schatzmayr H.G. (1990). Isolation of dengue virus type 2 in Rio de Janeiro. Mem. Inst. Oswaldo Cruz..

[8] Siqueira J.B., Vinhal L.C., Said R.F.C., Hoffmann J.L., Martins J., Barbiratto S.B. (2011). Dengue no Brasil: Tendências e mudanças na epidemiologia, com ênfase nas epidemias de 2008 e 2010. Saúde Brasil 010: Uma Análise da Situação de Saúde e de Evidências Selecionadas de Impacto de ações de Vigilância em Saúde.

[9] Rodriguez-Barraquer I., Cordeiro M.T., Braga C., Souza W.V., Marques E.T.T., Cummings D.A.T. (2011). From Re-Emergence to Hyperendemicity: The Natural History of the Dengue Epidemic in Brazil. PLoS Negl. Trop. Dis..

[10] Nunes P.C., Sampaio S.A., Rodrigues da Costa N., de Mendonça M.C., Lima M.D., Araujo S.E., dos Santos F.B., Simões J.B., de Santis Gonçalves B., Nogueira R.M. (2016). Dengue severity associated with age and a new lineage of dengue virus-type 2 during an outbreak in Rio De Janeiro, Brazil. J. Med. Virol..

[11] Torres M.C., Nogueira F.B., Fernandes C.A., Meira G.L.S., Aguiar S.F., Chieppe A.O., de Filippis A.M.B. (2019). Re-introduction of Dengue Virus Serotype 2 in the State of Rio De Janeiro After Almost a Decade of Epidemiological Silence. PLoS ONE.

[12] World Health Organization (2009). Dengue Guidelines for Diagnosis, Treatment, Prevention and Control.

[13] Jessie K., Fong M.Y., Devi S., Lam S.K., Wong K.T. (2004). Localization of dengue virus in naturally infected human tissues, by immunohistochemistry and in situ hybridization. J. Infect. Dis..

[14] Balsitis S.J., Coloma J., Castro G. (2009). Tropism of dengue virus in mice and humans defined by viral nonstructural protein 3-specific immunostaining. Am. J. Trop. Med. Hyg..

[15] Lima M.d.R., Nogueira R.M., Schatzmayr H.G., de Filippis A.M., Limonta D., dos Santos F.B. (2011). A new approach to dengue fatal cases diagnosis ns1 antigen capture in tissues. PLoS Negl. Trop. Dis..

[16] Rivera J.A., Rengifo A.C., Parra E.A., Castellanos J.E., Caldas M.L. (2020). Illustrated histopathological features of fatal dengue cases in Colombia. Histopatología ilustrada de casos fatales de dengue en Colombia. Biomedica.

[17] Póvoa T.F., Alves A.M., Oliveira C.A., Nuovo G.J., Chagas V.L., Paes M.V. (2014). The pathology of severe dengue in multiple organs of human fatal cases: Histopathology, ultrastructure and virus replication. PLoS ONE.

[18] Samanta J., Sharma V. (2015). Dengue and its effects on liver. World J. Clin. Cases.

[19] Pancharoen C., Rungsarannont A., Thisyakorn U. (2002). Hepatic dysfunction in dengue patients with various severity. J. Med. Assoc. Thail..

[20] Seneviratne S.L., Perera J. (2006). Fever epidemic moves into Sri Lanka. BMJ.

[21] Puccioni-Sohler M., Soares C.N., Papaiz-Alvarenga R., Castro M.J., Faria L.C., Peralta J.M. (2009). Neurologic dengue manifestations associated with intrathecal specific immune response. Neurology.

[22] Laoprasopwattana K., Pruekprasert P., Dissaneewate P., Geater A., Vachvanichsanong P. (2010). Outcome of dengue hemorrhagic fever-caused acute kidney injury in Thai children. J. Pediatr..

[23] Rojas E.M., Herrera V.M., Miranda M.C., Rojas D.P., Gómez A.M., Pallares C., Cobos S.M., Pardo L., Gélvez M., Páez A. (2019). Clinical Indicators of Fatal Dengue in Two Endemic Areas of Colombia: A Hospital-Based Case-Control Study. Am. J. Trop. Med. Hyg..

[24] Salomão N., Rabelo K., Basílio-de-Oliveira C., Basílio-de-Oliveira R., Geraldo L., Lima F., Dos Santos F., Nuovo G., Oliveira E., Paes M. (2020). Fatal Dengue Cases Reveal Brain Injury and Viral Replication in Brain-Resident Cells Associated with the Local Production of Pro-Inflammatory Mediators. Viruses.

[25] Diptyanusa A., Phumratanaprapin W. (2021). Predictors and Outcomes of Dengue-Associated Acute Kidney Injury. Am. J. Trop. Med. Hyg..

[26] Cunha M., Duarte-Neto A.N., Pour S.Z., Hajjar L.A., Frassetto F.P., Dolhnikoff M., Saldiva P., Zanotto P. (2021). Systemic dengue infection associated with a new dengue virus type 2 introduction in Brazil—A case report. BMC Infect. Dis..

[27] Nunes P., Rioja L., Coelho J., Salomão N.G., Rabelo K., José C.C., Rodrigues F., de Azeredo E.L., Basílio-de-Oliveira C.A., Basílio-de-Oliveira R. (2019). Renal Injury in DENV-4 Fatal Cases: Viremia, Immune Response and Cytokine Profile. Pathogens.

[28] Boonpucknavig V., Bhamarapravati N., Boonpucknavig S., Futrakul P., Tanpaichitr P. (1976). Glomerular changes in dengue hemorrhagic fever. Arch. Pathol. Lab. Med..

[29] Wiersinga W.J., Scheepstra C.G., Kasanardjo J.S., de Vries P.J., Zaaijer H., Geerlings S.E. (2006). Dengue fever–induced hemolytic uremic syndrome. Clin. Infect. Dis..

[30] Lima E.Q., Nogueira M.L. (2008). Viral hemorrhagic fever-induced acute kidney injury. Semin. Nephrol..

[31] Lim C.T.S., Fuah K.W., Lee S.E., Kaniappan K.K., Then R.F. (2019). Dengue-Associated Acute Kidney Infection: An Updated and Comprehensive Qualitative Review of Literature. EMJ Nephrol..

[32] Gulati S., Maheshwari A. (2007). Atypical manifestations of dengue. Trop. Med. Int. Health.

[33] Lizarraga K.J., Nayer A. (2014). Dengue-associated kidney disease. J. Nephropathol..

[34] Oliveira J.F., Burdmann E.A. (2015). Dengue-associated acute kidney injury. Clin. Kidney J..

[35] Vakrani G.P., Subramanyam N.T. (2017). Acute Renal Failure in Dengue Infection. J. Clin. Diagn. Res..

[36] Kuo M.C., Lu P.L., Chang J.M., Lin M.Y., Tsai J.J., Chen Y.H., Chang K., Chen H.C., Hwang S.J. (2008). Impact of renal failure on the outcome of dengue viral infection. Clin. J. Am. Soc. Nephrol. CJASN.

[37] Mallhi T.H., Khan A.H., Adnan A.S., Sarriff A., Khan Y.H., Jummaat F. (2015). Incidence, Characteristics and Risk Factors of Acute Kidney Injury among Dengue Patients: A Retrospective Analysis. PLoS ONE.

[38] Naqvi R., Mubarak M., Ahmed E., Akhtar F., Naqvi A., Rizvi A. (2016). Acute tubulointerstitial nephritis/drug induced acute kidney injury; an experience from a single center in Pakistan. J. Renalrenal Inj. Prev..

[39] Diptyanusa A., Phumratanaprapin W., Phonrat B., Poovorawan K., Hanboonkunupakarn B., Sriboonvorakul N., Thisyakorn U. (2019). Characteristics and associated factors of acute kidney injury among adult dengue patients: A retrospective single-center study. PLoS ONE.

[40] Eswarappa M., Reddy S.B., John M.M., Suryadevara S., Madhyashatha R.P. (2019). Renal manifestations of dengue viral infection. Saudi J. Kidney Dis. Transpl..

[41] Prasad N., Novak J.E., Patel M.R. (2019). Kidney Diseases Associated with Parvovirus B19, Hanta, Ebola, and Dengue Virus Infection: A Brief Review. Adv. Chronic Kidney Dis..

[42] Repizo L.P., Malheiros D.M., Yu L., Barros R.T., Burdmann E.A. (2014). Biopsy proven acute tubular necrosis due to rhabdomyolysis in a dengue fever patient: A case report and review of literature. Rev. Inst. Med. Trop. Sao Paulo.

[43] Pagliari C., Simões Quaresma J.A., Kanashiro-Galo L., de Carvalho L.V., Vitoria W.O., da Silva W.L., Penny R., Vasconcelos B.C., da Costa Vasconcelos P.F., Duarte M.I. (2016). Human kidney damage in fatal dengue hemorrhagic fever results of glomeruli injury mainly induced by IL17. J. Clin. Virol..

[44] Póvoa T.F., Oliveira E.R.A., Basílio-de-Oliveira C.A., Nuovo G.J., Chagas V.L.A., Salomão N.G., Alves A.M.B., Mota E.M., Paes M.V. (2018). Correction: Peripheral Organs of Dengue Fatal Cases Present Strong Pro-Inflammatory Response with Participation of IFN-Gamma, TNF-Alpha- and RANTES-Producing Cells. PLoS ONE.

[45] Guzman M.G., Harris E. (2015). Dengue. Lancet.

[46] Martina B.E., Koraka P., Osterhaus A.D. (2009). Dengue virus pathogenesis: An integrated view. Clin. Microbiol. Rev..

[47] Halstead S.B. (2015). Pathogenesis of Dengue: Dawn of a New Era. F1000Research.

[48] Huy N.T., Thao N.T., Ha T.T., Lan N.T., Nga P.T., Thuy T.T., Tuan H.M., Nga C.T., Tuong V.V., Dat T.V. (2013). Development of clinical decision rules to predict recurrent shock in dengue. Crit. Care.

[49] Carabali M., Hernandez L.M., Arauz M.J., Villar L.A., Ridde V. (2015). Why are people with dengue dying? A scoping review of determinants for dengue mortality. BMC Infect. Dis..

[50] Chan K.W.K.S., Watanabe R., Kavishna S., Alonso S., Vasudevan S.G. (2015). Animal models for studying dengue pathogenesis and therapy. Antivir. Res..

[51] Rico-Hesse R., Harrison L., Salas R., Tovar D., Nisalak A., Ramos C., Boshell J., de Mesa M.T., Nogueira R.M., da Rosa A.T. (1997). Origins of dengue type 2 viruses associated with increased pathogenicity in the Americas. Virology.

[52] Zompi S., Harris E. (2012). Animal models of dengue virus infection. Viruses.

[53] Oliveira E.R., Amorim J.F., Paes M.V., Azevedo A.S., Gonçalves A.J., Costa S.M., Mantuano-Barradas M., Póvoa T.F., de Meis J., Basílio-de-Oliveira C.A. (2016). Peripheral effects induced in BALB/c mice infected with DENV by the intracerebral route. Virology.

[54] Atrasheuskaya A., Petzelbauer P., Fredeking T.M., Ignatyev G. (2003). Anti-TNF antibody treatment reduces mortality in experimental dengue virus infection. FEMS Immunol. Med. Microbiol..

[55] Paes M.V., Pinhão A.T., Barreto D.F., Costa S.M., Oliveira M.P., Nogueira A.C., Takiya C.M., Farias-Filho J.C., Schatzmayr H.G., Alves A.M. (2005). Liver injury and viremia in mice infected with dengue-2 virus. Virology.

[56] Paes M.V., Lenzi H.L., Nogueira A.C., Nuovo G.J., Pinhão A.T., Mota E.M., Basílio-de-Oliveira C.A., Schatzmayr H., Barth O.M., Alves A.M. (2009). Hepatic damage associated with dengue-2 virus replication in liver cells of BALB/c mice. Lab. Investig..

[57] França R.F., Zucoloto S., da Fonseca B.A. (2010). A BALB/c mouse model shows that liver involvement in dengue disease is immune-mediated. Exp. Mol. Pathol..

[58] Barreto D.F., Takiya C.M., Paes M.V., Farias-Filho J., Pinhão A.T., Alves A.M., Costa S.M., Barth O.M. (2004). Histopathological aspects of Dengue-2 virus infected mice tissues and complementary virus isolation. J. Submicrosc. Cytol. Pathol..

[59] Tuiskunen A., Wahlström M., Bergström J., Buchy P., Leparc-Goffart I., Lundkvist A. (2011). Phenotypic characterization of patient dengue virus isolates in BALB/c mice differentiates dengue fever and dengue hemorrhagic fever from dengue shock syndrome. Virol. J..

[60] Sakinah S., Priya S.P., Kumari S., Amira F., Poorani K., Alsaeedy H., Ling M.P., Chee H.Y., Higuchi A., Alarfaj A.A. (2017). Impact of dengue virus [serotype DENV-2] infection on liver of BALB/c mice: A histopathological analysis. Tissue Cell.

[61] Rasinhas A.D.C., Silva M.A.N.D., Caldas G.C., Jácome F.C., Leonardo R., Santos F.B.D., Nunes P.C.G., Barth O.M., Barreto-Vieira D.F. (2018). First detection of dengue virus in the saliva of immunocompetent murine model. Mem. Inst. Oswaldo Cruz..

[62] Salomão N.G., Rabelo K., Póvoa T.F., Alves A., da Costa S.M., Gonçalves A., Amorim J.F., Azevedo A.S., Nunes P., Basílio-de-Oliveira C.A. (2018). BALB/c mice infected with DENV-2 strain 66985 by the intravenous route display injury in the central nervous system. Sci. Rep..

[63] Jácome F.C., Caldas G.C., Rasinhas A., de Almeida A., de Souza D., Paulino A.C., Leonardo R., Barth O.M., Dos Santos F.B., Barreto-Vieira D.F. (2021). Comparative analysis of liver involvement caused by two DENV-2 lineages using an immunocompetent murine model. Sci Rep..

[64] Nogueira R.M.R., Araújo J.M.G., Schatzmayr H.G. (2007). Dengue viroses in Brazil, 1986–2006. Rev. Panam. Salud Publica.

[65] Dissanayake H.A., Seneviratne S.L. (2018). Liver involvement in dengue viral infections. Rev. Med. Virol..

[66] Acharya S., Shukla S., Mahajan S.N., Diwan S.K. (2010). Acute dengue myositis with rhabdomyolysis and acute renal failure. Ann. Indian Acad. Neurol..

[67] Bhagat M., Zaki S.A., Sharma S., Manglani M.V. (2012). Acute glomerulonephritis in dengue haemorrhagic fever in the absence of shock, sepsis, haemolysis or rhabdomyolysis. Paediatr. Int. Child Health.

[68] Mehra N., Patel A., Abraham G., Reddy Y.N., Reddy Y.N. (2012). Acute kidney injury in dengue fever using Acute Kidney Injury Network criteria: Incidence and risk factors. Trop. Dr..

[69] Vachvanichsanong P., Thisyakorn U., Thisyakorn C. (2016). Dengue hemorrhagic fever and the kidney. Arch. Virol..

[70] Setiawan M.W., Samsi T.K., Wulur H., Sugianto D., Pool T.N. (1998). Dengue haemorrhagic fever: Ultrasound as an aid to predict the severity of the disease. Pediatr. Radiol..

[71] Arshad K., Sheikh S., Naqvi S.U., Sarwar I., Javaid S., Asghar M., Butt M.A. (2015). Frequency of splenomegaly in dengue fever in children. J. Ayub Med. Coll. Abbottabad.

[72] Fernando S., Wijewickrama A., Gomes L., Punchihewa C.T., Madusanka S.D., Dissanayake H., Jeewandara C., Peiris H., Ogg G.S., Malavige G.N. (2016). Patterns and causes of liver involvement in acute dengue infection. BMC Infect. Dis..

[73] Ferreira R., Kubelka C.F., Velarde L., Matos J., Ferreira L.C., Reid M.M., Setúbal S., Oliveira S.A. (2018). Predictive factors of dengue severity in hospitalized children and adolescents in Rio de Janeiro, Brazil. Rev. Soc. Bras. Med. Trop..

[74] Caldas G.C. (2019). Modelo Murino Imunocompetente para Estudo da Infecção pelo vírus Dengue 3: Aspectos Morfológicos, Viremia e Tropismo. Master’s Thesis.

[75] Basílio-de-Oliveira C.A., Aguiar G.R., Baldanza M.S., Barth O.M., Eyer-Silva W.A., Paes M.V. (2005). Pathologic Study of a Fatal Case of Dengue-3 Virus Infection in Rio de Janeiro, Brazil. Braz. J. Infect. Dis..

[76] Mohsin N., Mohamed E., Gaber M., Obaidani I., Budruddin M., Al Busaidy S. (2009). Acute tubular necrosis associated with non-hemorrhagic Dengue fever: A case report. Ren. Fail..

[77] Tansir G., Gupta C., Mehta S., Kumar P., Soneja M., Biswas A. (2017). Expanded dengue syndrome in secondary dengue infection: A case of biopsy proven rhabdomyolysis induced acute kidney injury with intracranial and intraorbital bleeds. Intract. Rare Dis. Res..

[78] Queiroz P.C., Jorge A., Mourão P., Penido M. (2020). Collapsing focal segmental glomerulosclerosis probably triggered by dengue virus infection—two case reports. J. Bras. Nefrol..

[79] Vasanwala F.F., Thein T.L., Leo Y.S., Gan V.C., Hao Y., Lee L.K., Lye D.C. (2014). Predictive value of proteinuria in adult dengue severity. PLoS Negl. Trop. Dis..

[80] Boonpucknavig V., Soontornniyomkij V. (2003). Pathology of renal diseases in the tropics. Semin. Nephrol..

[81] Upadhaya B.K., Sharma A., Khaira A., Dinda A.K., Agarwal S.K., Tiwari S.C. (2010). Transient IgA nephropathy with acute kidney injury in a patient with dengue fever. Saudi J. Kidney Dis. Transpl..

[82] Basu A., Chaturvedi U.C. (2008). Vascular endothelium: The battlefield of dengue viruses. FEMS Immunol. Med. Microbiol..

[83] Gubler D.J. (1998). Dengue and dengue hemorrhagic fever. Clin. Microbiol. Rev..

[84] Puerta-Guardo H., Glasner D.R., Harris E. (2016). Dengue Virus NS1 Disrupts the Endothelial Glycocalyx, Leading to Hyperpermeability. PLoS Pathog..

[85] Aye K.S., Charngkaew K., Win N., Wai K.Z., Moe K., Punyadee N., Thiemmeca S., Suttitheptumrong A., Sukpanichnant S., Prida M. (2014). Pathologic highlights of dengue hemorrhagic fever in 13 autopsy cases from Myanmar. Hum. Pathol..

[86] Malavige G.N., Ogg G.S. (2017). Pathogenesis of vascular leak in dengue virus infection. Immunology.

[87] Limonta D., Falcón V., Torres G., Capó V., Menéndez I., Rosario D., Castellanos Y., Alvarez M., Rodríguez-Roche R., de la Rosa M.C. (2012). Dengue virus identification by transmission electron microscopy and molecular methods in fatal dengue hemorrhagic fever. Infection.

[88] Wiwanitkit V. (2005). Acute renal failure in the fatal cases of dengue hemorrhagic fever, a summary in Thai death cases. Ren. Fail..

[89] Lee I.K., Liu J.W., Yang K.D. (2009). Clinical characteristics, risk factors, and outcomes in adults experiencing dengue hemorrhagic fever complicated with acute renal failure. Am. J. Trop Med. Hyg..

[90] Moi M.L., Omatsu T., Hirayama T., Nakamura S., Katakai Y., Yoshida T., Saito A., Tajima S., Ito M., Takasaki T. (2013). Presence of Viral Genome in Urine and Development of Hematuria and Pathological Changes in Kidneys in Common Marmoset [*Callithrix jacchus*] after Inoculation with Dengue Virus. Pathogens.

[91] Wu S.J., Hayes C.G., Dubois D.R., Windheuser M.G., Kang Y.H., Watts D.M., Sieckmann D.G. (1995). Evaluation of the severe combined immunodeficient [SCID] mouse as an animal model for dengue viral infection. Am. J. Trop. Med. Hyg..

[92] Davis J.S., Bourke P. (2004). Rhabdomyolysis associated with dengue virus infection. Clin. Infect. Dis..

[93] Wijesinghe A., Gnanapragash N., Ranasinghe G., Ragunathan M.K. (2013). Acute renal failure due to rhabdomyolysis following dengue viral infection: A case report. J. Med. Case Rep..

[94] Kamath N., Iyengar A. (2018). Infections and the kidney: A tale from the tropics. Pediatr. Nephrol..

[95] Gubler D.J., Kuno G., Sather G.E., Velez M., Oliver A. (1984). Mosquito cell cultures and specific monoclonal antibodies in surveillance for dengue viruses. Am. J. Trop. Med. Hyg..

[96] Lanciotti R.S., Calisher C.H., Gubler D.J., Chang G.J., Vorndam A.V. (1992). Rapid detection and typing of dengue viruses from clinical samples by using reverse transcriptase-polymerase chain reaction. J. Clin. Microbiol..

[97] Reed L.J., Muench H. (1938). A simple method of estimating fifty percent endpoints. Am. J. Epidemiol..

[98] Barreto D.F., Barth M.O., Schatzmayr H.G. (2010). Modelo Animal Experimental Para o Estudo da Patogênese dos Vírus Dengue Sorotipos 1 e 2.

